# English learning anxiety of Chinese rural junior high school students under the online class mode

**DOI:** 10.3389/fpsyg.2023.1156092

**Published:** 2023-06-23

**Authors:** Qiangfu Yu, Dazhou Xu, Rui Huang

**Affiliations:** Faculty of Humanities and Foreign Languages, Xi’an University of Technology, Xi’an, Shaanxi, China

**Keywords:** online class mode, Chinese rural junior high school students, English learning anxiety, online learning, foreign language learning anxiety

## Abstract

The COVID-19 pandemic has brought tremendous changes to the field of education, transferring traditional offline teaching to online teaching on a large scale globally. Junior high school students, as a special group, may experience foreign language learning anxiety different from anxiety experienced by college students in the process of online English learning. This research aims at investigating the level of, sources and strategies for English learning anxiety of Chinese rural junior high school students under online class mode. A total of 120 students from Dongshan Junior High School in Haikou participated in this study and asked to fill in the questionnaires, and 12 of them were randomly chosen to be interviewed. IBM SPSS Statistics 26 was used to analyze the data. This research found that Chinese rural junior high school students generally had a moderate level of English learning anxiety, and there is statistically no significant relation between the gender difference and anxiety in online foreign language classes. It was also found that factors influencing English learning anxiety of Chinese rural junior high school students included the students themselves, their home environments, the teacher and the school, and the social environment. Lastly, the research found five strategies to relieve foreign language learning anxiety, including recognizing the existence of anxiety correctly, communicating the anxiety honestly with others, improving the psychological quality, being positive about life’ s setbacks, and setting up some realistic goals in English learning.

## Introduction

Since the 1950s, with the development of humanistic psychology, emotional factors began to receive widespread attention from scholars (Chastain, 1975; Tsai and Lee, 2018; Pawlak et al., 2020). Foreign language learning anxiety, as an important affective factor influencing learners’ learning efficiency, has attracted wide attention (Horwitz et al., 1986; Liu and Jackson, 2008; Marcos-Llinás and Garau, 2009; Dewaele and Mac Intyre, 2014, 2016; Saghafi et al., 2017; Boudreau et al., 2018; Elahi Shirvan and Talebzadeh, 2018a, 2020; Dewaele et al., 2019; Mac Intyre et al., 2019; Elahi Shirvan and Taherian, 2021). In today’s global pandemic environment, junior high school students under the online class mode, as a special group, may experience foreign language learning anxiety different from the anxiety experienced by college students in the process of English learning (Dewaele and Dewaele, 2017; Li, 2020). However, previous studies are mainly focused on college students (Shi and Fan, 2013; Meng and Chen, 2014; Elahi Shirvan and Talebzadeh, 2018b; Wang and Zhang, 2021; Fong et al., 2022), and there are few studies on foreign language learning anxiety of junior high school students (Yu, 2022), and fewer on English learning anxiety of rural junior high school students under the online class mode. Therefore, this study investigates the level of and sources influencing English learning anxiety of Chinese rural junior high school students under online class mode, and explores the relationship between gender difference and online foreign language learning anxiety and its strategies.

## Literature review

### Definitions of foreign language learning anxiety

By the 1970s, foreign language learning anxiety began to receive a higher degree of attention. A major breakthrough in the study of foreign language learning anxiety began in the 1980s, when Horwitz (1986) and his colleagues first introduced the concept of foreign language learning anxiety in 1986. Horwitz et al. (1986, pp. 125–132) defined foreign language anxiety (FLA) as “a distinct complex of self-perceptions, beliefs, feelings, and behaviors related to classroom language learning arising from the uniqueness of the language learning process.” Since then, different researchers have given their own understanding of the connotation of foreign language learning anxiety from their own research standpoint. In recent years, foreign language learning anxiety was regarded as a kind of psychological state that learners show when they engage in foreign language communication activities (Li and Li, 2016) or an emotion in a fluctuating state over time (Saghafi and Elahi Shirvan, 2020).

### Factors and strategies for foreign language learning anxiety

So far, a variety of independent variables are found to be associated with foreign language learning anxiety. As early as 1990s, Saito et al. (1999) found that the foreign language reading anxiety of learners was negatively correlated with their academic performance, and reading anxiety. In recent years, scholars have added multiple pressures (Shi and Xu, 2013), contextual factors (Gkonou, 2017; Saghafi et al., 2017), self-efficacy (Zhang and Guo, 2018), learning strategies (Liu, 2020) and other variables when discussing the relationship between foreign language learning anxiety and foreign language achievement. Besides, Garcia de Blakeley et al. (2017) found students’ FLA was not affected by their gender. However, according to Dewaele et al. (2019), male Kazakh Turkish language learners have a higher FLA level in the classroom than their female peers. Therefore, the effect of gender on FLA has not reached a consensus.

On the basis of exploring the causes of foreign language learning anxiety, it has been an important theme for researchers to further explore the strategies to relieve it. Miyazoe and Anderson (2011) proved in an experimental study that the level of foreign language writing anxiety of students under the mode of anonymously publishing essays online was significantly lower than that under face-to-face guidance. Fong et al. (2022) examined speech anxiety among Malaysian Chinese college students at a private university, and found that rehearsing and practicing before giving a speech was an effective strategy for dealing with speech anxiety.

### Research on English learning anxiety under the online class mode

Due to the impact of the pandemic in the past 3 years, foreign language learning anxiety through the Internet has attracted more attention from scholars at home and abroad. For example, Russell (2020) examined language anxiety in the context of an online learning environment. Then, Li and Han (2022) conducted a questionnaire survey on the levels of pleasure, anxiety and boredom of 348 Chinese non-English majors in online foreign language classes and the relationships among them, and investigated the independent and synergistic predictive effects of these three emotions on the learning outcomes of online foreign language courses.

### Research on English learning anxiety of Chinese rural students

Much less attention has been paid to the online learning experience of EFL high school students, especially those from rural backgrounds. Wang (2015) put forward five strategies to alleviate rural high students’ English learning anxiety through qualitative analysis. Deng et al. (2022) examined students’ anxiety in medical English vocabulary, listening and speaking, communication, literature reading, and academic paper writing, and found the anxiety of rural medical students in each dimension was higher than that of urban medical students.

### Foreign language learning anxiety scales

In 1986, Horwitz et al. designed the Foreign Language Classroom Anxiety Scale (FLCAS), which mainly included three aspects of foreign language learning anxiety, namely, communicative fear, test anxiety, and negative evaluation fear. In 1991, Horwitz showed that the scale had high reliability, validity and internal consistency through several tests of the Foreign Language Classroom Anxiety Inventory. Since then, many scholars have done a large number of theoretical and practical research using this scale (Aida, 1994; Liu and Huang, 2011; Piniel and Csizér, 2015; Boudreau et al., 2018; Fong et al., 2022).

Previous studies on the relationship between anxiety and foreign language learning performance cover a wide range of aspects, including age, listening, reading, writing, and foreign language. However, the subjects of foreign language anxiety research mainly focus on undergraduate students, a small amount of research focuses on graduate students and international students, and few studies involve primary and secondary school students. In addition, the effect of gender on FLA has not reached a consensus. What’s more, there are even fewer studies focusing on English learning anxiety of junior high school students in the online class mode. Therefore, this study aims to investigate the level of and factors influencing English learning anxiety of Chinese rural junior high school students under online class mode, and to explore the relationship between gender difference and online foreign language learning anxiety and its strategies, enriching the content and perspective of foreign language learning anxiety research under the online class mode.

## Research design

### Research questions

This research aims to answer the following questions:

1. What is the overall situation of Chinese rural junior high school students under the online class mode?
2. Is there any difference between male and female students in this respect?
3. What are the factors influencing online foreign language anxiety for Chinese rural junior high school students?
4. What are the strategies to relieve online foreign language anxiety for Chinese rural junior high school students?

### Research methodology

Mixed method research is a reliable way to integrate qualitative and quantitative research, and more inference can be drawn from a study using mixed method approaches than from a study using only quantitative or qualitative approaches (Guetterman, 2017). Therefore, the research was divided into two stages: the first stage was quantitative research, and the second stage was carried out after obtaining specific data, and the data of the first stage were further interpreted through qualitative research (Creswell et al., 2003).

Quantitative research includes designing issuing, collecting questionnaires, and analyzing the data with IBM SPSS Statistics 26.0. Questionnaires were distributed by sharing QR codes and questionnaire website links with respondents. Respondents can fill out questionnaires online via mobile phone or computer. They are required to fill in the questionnaire in an anonymous manner according to their real situation.

Qualitative research includes semi-structured interview to improve the scientificity and objectivity of the research, because anxiety is an internal psychological state, there are certain differences in the thoughts of each person and the answers in the questionnaire are fixed, which cannot accurately describe the emotional state and anxiety status of students, resulting in the survey results are not completely accurate.

### Research participants

120 students were randomly selected as the participants of the research from the Dongshan Junior High School in Dongshan Town, Xiuying District, Haikou City, Hainan Province. All participants were in the first year of junior high school, with their ages ranging between 11 and 13 years old. These students had 1 year of online course learning experience and the same or similar characteristics (for all of them were from rural areas in Haikou and came from the rural junior high school), which made the research participants, to a large extent, represent Chinese junior high school students in rural areas. And through analyzing English learning anxiety of the participants under the online class mode, this study can catch a glimpse of the current situation of English learning anxiety of Chinese rural junior high school students under the online class mode.

### Research instruments

The study mainly adopts questionnaires to explore English learning anxiety of junior high school students under the online class mode. In order to ensure the accuracy of the research, the interview method is also adopted.

### Questionnaire based on foreign language classroom anxiety scale

The questionnaire consists of 45 questions and is divided into two parts. The first 10 questions are about the socio-demographic information of the subjects; the last 35 questions are adapted from Foreign Language Classroom Anxiety Scale (FLCAS) designed by Horwitz (1986) and Chinese version of Horwitz’ FLCAS translated by Wang (2003). The 35 questions of the questionnaire can be divided into four dimensions: communicative anxiety includes 10 questions (Q11, Q15, Q18, Q19, Q33, Q34, Q37, Q42, Q43, and Q45); test anxiety includes 5 questions (Q17, Q24, Q25, Q31, and Q44); negative evaluation anxiety includes 9 questions, (Q16, Q20, Q22, Q26, Q27, Q32, Q39, Q40, and Q41); online class anxiety includes 11 questions, (Q12, Q13, Q14, Q21, Q23, Q28, Q29, Q30, Q35, Q36, and Q38). Each question was followed by five options, 1 = not at all in my situation; 2 = not quite in my situation; 3 = sometimes in my situation; 4 = more in my situation; 5 = completely in my situation. The subjects were asked to select the one that best fits their situation from the five options. The scoring statistics were scored using a five-point Likert scale, i.e., 1 = this practice does not fit me at all or almost at all; 2 = this practice does not fit me most of the time; 3 = this practice fits me mostly; 4 = this practice fits me most of the time; 5 = this practice fits me completely or almost completely. Higher scores indicate higher levels of English learning anxiety of junior high school students under the online class mode. Thus, the total anxiety scores fluctuate between 35 and 175.

Before applying this questionnaire formally, the reliability and validity of the questionnaire was tested, and the analysis results are shown in Table 1. As can be seen from the table, the overall Cronbach’s alpha value of the adapted questionnaire is 0.904. Among them, the Cronbach’s alpha values of the four dimensions of the adapted questionnaire (communicative anxiety, test anxiety, negative evaluation anxiety and online class anxiety) are 0.743, 0.718, 0.804, and 0.851 (0.7 < α < 0.8), indicating that questionnaire had a high reliability. A validity analysis then was performed on the questionnaire. As can be seen from the Table 2, KMO is 0.813and the approximate chi-square value for the Bartlett spherical test is 2019.552, with a significance level of standard (*p* = 0.000), which indicated that the questionnaire has high validity.

**Table 1:** Scale reliability analysis.

Dimension	Cronbach’s alpha value	Number of items
communicative anxiety,	0.743	10
test anxiety	0.718	5
negative evaluation anxiety	0.804	9
online class anxiety	0.851	11
Scale	0.904	35

**Table 2:** Scale validity analysis.

Statistics of KMO and Bartlett’ s test		
Kaiser-Meyer-Olkin measure of sampling adequacy.		0.813
Bartlett’ s test of sphericity	Approx. Chi-Square	2019.552
	df	595
	Sig.	0.000

### Semi-structured interview

Semi-structured interview guidance questions were used in the interview. The authors developed four main questions referring to the foreign language anxiety interview guidance questions in the study by Zhang (2012), aiming at detecting the anxiety of rural junior high school students in online foreign language classes. They are as follows:

1. Do you feel anxious when learning English in the mode of online class? If so, when do you feel anxious?
2. How do you think English learning anxiety affects your English study performance?
3. What do you think are the factors that affect your English learning anxiety?
4. What are your suggestions for reducing English learning anxiety?

The first interview question in this study was explored to determine whether online foreign language anxiety exists in rural junior high school. The second question was aimed to collect the influence of anxiety on rural junior high school students’ English learning. The third interview question was focused on the influencing factors of anxiety in online foreign language. The fourth question was aimed to find strategies to alleviate this anxiety.

The reliability of the interview data was enhanced in the following ways. First of all, the interview group was made up of a female teacher with 8 years of English teaching experience and a male teacher with 14 years of English teaching experience, who had similar explanations and views on the performance of the interviewees. Secondly, interview techniques such as a carry-on feedback and an encouraging elaboration expounded by Zoltán (2007) were used to make the interview process more flexible. Thirdly, interviewers adjusted questioning ways according to the interviewees’ personality and performance, so that the interview process would be more natural. Fourth, individual interviews were conducted in Chinese instead of English, so that students could express their ideas more clearly in their mother tongue.

### Data collection

After the design of the present study was approved by the Research Committee of the department and the school, the data were collected between the 9^th^ week and 11^th^ week in the 20-week autumn semester, 2022. The questionnaire and a consent form were distributed online to the 120 students, which yielded 112 valid questionnaires. Of the 8 invalid questionnaires, 5 were completed in less than 180 s, and 3 were completed with the same choices for all the questions.

Among the valid questionnaires obtained, 48 (42.86%) were from male students and 64 (57.14%) were from female students. In terms of their scores, interviewees included, 2 failed male students and 2 failed female students (with a score range of 35–81), 2 average male students and 2 average female students (with a score range of 82–128), and 2 top male students and 2 top female students (with a score range of 129–175). Therefore, with an even distribution of genders, the interviewees could show the current situation of English learning anxiety of junior high school students under the online class mode. The interviews were conducted by way of Wechat video chat, which were recorded for future analysis, the duration of interview for each person being controlled to 15–20 min. By average, each interview lasted 16.91 min. Due to the low level of English proficiency of the students, the interviews were conducted in the language of Mandarin Chinese. During the interview, mobile phones were used to record, and then the interview conversations were transcribed into notes, which were classified, analyzed and summarized. The quotations concerning the interviewees’ words in the section of Results and Discussion were translated from Chinese to English by the correspondence author, an EFL teacher with 15 years of teaching and translation practice.

### Data analysis

The data base was created with Excel software, and the IBM SPSS Statistics 26 statistical analysis package was used to analyze the data. Mean and standard deviation were calculated for each dimension and question to obtain the general level of English learning anxiety of junior high school students under the online class mode. The independent sample t-test, mean and standard deviation were carried out on the relation of gender and anxiety about online foreign language classes to solve the second research question. Mean and standard deviation were used to explore the factors and strategies for online foreign language anxiety to address the third and last research objective.

The analysis of interview data included four steps. Firstly, all interviews were transcribed and translated into English by the authors. Furthermore, the transcribed and translated texts were double-checked, during which the authors sought validation from respondents through smartphone conversations when ambiguities arose. Then the transcripts were precoded and encoded to make the information structured and manageable (Chen, 2000). Next, the coded text was analyzed and the ideas-memos were taken down. On the basis of the analysis, various specific factors affecting foreign language anxiety and strategies for online foreign language anxiety were finally classified and statistically analyzed.

## Results and discussion

### General level of foreign language learning anxiety for rural junior high school students

In order to explore the overall level of students’ foreign language anxiety, students were divided into high anxiety group, moderate anxiety group, and low anxiety group according to their total score of anxiety and the average score of the four dimensions. The students were classified as a high anxiety group with a higher score of one standard deviation, and the students were classified as a low anxiety group with a lower score of one standard difference. The remaining students were classified as moderate anxiety group (Table 3).

**Table 3:** Scale score.

Anxiety	M ± SD
Communicative anxiety	2.659 ± 0.741
Test anxiety	2.536 ± 0.828
Negative evaluation anxiety	2.413 ± 0.821
Online class anxiety	2.583 ± 0.603
Overall	2.496 ± 0.795

As shown in Table 4, 77.68% of the students surveyed had a moderate score and above. Among all dimensions, students with communicative anxiety accounted for the highest proportion (83.92%), followed by test anxiety (81.52%), negative evaluation anxiety (80.63%), and online class anxiety (79.47%).

**Table 4:** Distribution of samples at each latitude.

	Communicative anxiety		Negative evaluation anxiety		Test anxiety		Online class anxiety		Total anxiety score	
	N.	Percentage	N.	Percentage	N.	Percentage	N.	Percentage	N.	Percentage
High anxiety	26	23.21%	36	32.14%	27	24.11%	31	27.68%	25	22.32%
Moderate anxiety	68	60.71%	55	49.11%	63	56.25%	58	51.79%	62	55.36%
Low anxiety	18	16.07%	21	18.75%	22	19.64%	23	20.54%	25	22.32%

### Communicative anxiety

The highest mean value of 3.04 can be seen in Table 5 for question Q19, which indicates that junior high school students have a fearful mindset when speaking in online English class, especially when they are not prepared.

**Table 5:** Descriptive statistics of each question about communicative anxiety.

Title	Mean	Gender	N.	Mean	SD	T	DF	Sig.
Q11:I consider myself an extrovert	2.87	M	48	3.06	1.359	1.316	110	0.191
		F	64	2.72	1.374			
Q15:Speaking English to the screen during online classes makes me feel embarrassed	2.56	M	48	2.73	1.380	1.135	110	0.259
		F	64	2.44	1.320			
Q18:I feel nervous when the signal is bad or the English teacher asks me questions during online classes, whether I am prepared or not	2.89	M	48	2.88	1.424	−0.112	110	0.911
		F	64	2.91	1.488			
Q19:I feel very nervous when I am called on to answer a question during the Internet class	3.04	M	48	3.15	1.353	0.662	110	0.509
		F	64	2.97	1.436			
Q33:When I learn English in online classes, I do not dare to ask the teacher, I do not have classmates around me, and I cannot find a solution to the problems I do not know.	2.62	M	48	2.83	1.449	1.521	110	0.131
		F	64	2.45	1.194			
Q34:When the English teacher’s online classes are fast-paced, I worry that I will not be able to keep up	2.69	M	48	2.83	1.492	0.978	87.083	0.331
		F	64	2.58	1.179			
Q37:I will participate in as few online English activities as possible for fear of embarrassment	2.17	M	48	2.35	1.329	1.304	110	0.195
		F	64	2.03	1.272			
Q42:Due to the pandemic, it was difficult to go out, which reduced my usual exposure to and learning of English	2.43	M	48	2.54	1.501	0.743	89.715	0.459
		F	64	2.34	1.237			
Q43:In the online class mode, there was no sense of the usual atmosphere of reading words and texts together as a class, and the pace of learning was disrupted, which made me feel annoyed.	2.39	M	48	2.42	1.302	0.168	110	0.867
		F	64	2.38	1.303			
Q45:Compared with traditional classroom teaching, I feel more relaxed and fluent in English when I answer questions in English during online classes	2.93	M	48	2.83	1.374	−0.656	110	0.513
		F	64	3.00	1.297			
Communicative anxiety	2.66	M	48	2.763	0.751	1.285	110	0.202

The interviews with the students also prove that Chinese rural junior high school students have a moderate level of communicative anxiety in English learning. In interviews, Interviewee A said, “I am always uneasy, nervous and even panic when the teacher asks me to answer a question, because of my poor preparation.” Similarly, Interviewee D attributed her nervousness to “I do not make good preparation and sometimes I misunderstand the teacher’s questions.” Besides, Interviewee G said, “when communicating with the teacher, I always feel nervous and even overwhelmed because of my mistake.” The questionnaire also confirms this situation.

### Negative evaluation anxiety

The overall mean value of the students’ negative evaluation anxiety is 2.41, shown in Table 6. It reveals that students have a relatively low level of negative evaluation anxiety in English learning under the online class mode.

**Table 6:** Descriptive statistics of each question about negative evaluation anxiety.

Title	Mean	Gender	N.	Mean	SD	T	DF	Sig.
Q16:I’m especially worried about the damage to my eyes from online learning	2.29	M	48	2.54	1.473	1.677	110	0.096
		F	64	2.09	1.342			
Q20:I feel anxious when I do not understand what the teacher says or means in the Internet class	2.94	M	48	3.00	1.288	0.438	110	0.662
		F	64	2.89	1.323			
Q22:I get so nervous in English class that I forget everything I know	2.20	M	48	2.56	1.303	2.826	86.049	0.006
		F	64	1.92	1.013			
Q26:I am more nervous about taking English online classes than other subjects because of my poor foundation	2.43	M	48	2.67	1.464	1.904	110	0.060
		F	64	2.17	1.279			
Q27:I feel nervous and uneasy when there is a word I cannot understand when the signal is bad during online classes or when the English teacher says something	2.41	M	48	2.46	1.383	0.199	110	0.843
		F	64	2.41	1.365			
Q32:I feel very uneasy when the teacher corrects my mistakes or mentions something related to me in class	2.39	M	48	2.48	1.321	0.570	110	0.570
		F	64	2.34	1.185			
Q39:Most teachers try to teach online for the first time and the teaching effect is very poor	2.29	M	48	2.60	1.364	2.274	110	0.025
		F	64	2.06	1.153			
Q40:During online classes, I can only hear the teacher’s voice, and I do not have a good experience in class	2.33	M	48	2.44	1.287	0.764	110	0.447
		F	64	2.25	1.285			
Q41:Teachers cannot tell how well students are learning and cannot teach them according to their needs	2.46	M	48	2.60	1.440	0.987	110	0.326
		F	64	2.34	1.336			
Negative evaluation anxiety	2.41	M	48	2.595	0.743	2.064	110	0.041
		F	64	2.276	0.855			

The mean value of question Q20 is higher, followed by question Q41, and again by questions Q26 and Q27, shown in Table 6. This indicates that students are also still afraid of making mistakes when answering questions for fear of being ridiculed.

The interviews with the students also support the statistical results. Interviewee B said, “I am unwilling to answer the teacher’s questions, because I am afraid of being criticized by the teacher and laughed at by the students for giving wrong answers.” And Interviewee E mentioned in his interview, “I am reluctant to answer the teacher’s questions, because I think my English is not as good as other students’ English.” Similarly, Interviewee C said, “the teacher’s fluent spoken English and smooth speed make me unable to keep up with the teaching contents and unable to concentrate on the lessons, so I’m always very anxious in online English class.”

From the above interviews, we can clearly see that students are anxious about negative evaluation when learning English online, and they are not confident when answering questions, afraid of making mistakes, and afraid of being ridiculed.

### Test anxiety

Of all the questionnaire questions on test anxiety, Q24 has the highest mean value of 2.92 (Table 7). This indicates that students are more concerned about the consequences of failing an English test. This is followed by being nervous about some quizzes in English classes. This indicates that students are still concerned about exams, whether they are quizzes or large exams.

**Table 7:** Descriptive statistics of each question about test anxiety.

Title	Mean	Gender	N.	Mean	SD	T	DF	Sig.
Q17:I am very resistant to the online learning mode of online classes	2.06	M	48	2.19	1.363	0.918	110	0.361
		F	64	1.97	1.154			
Q24:I often worry that I will fail in English	2.92	M	48	3.02	1.550	0.623	110	0.534
		F	64	2.84	1.439			
Q25:I feel unsure of myself when I get close to a quiz or exam	2.66	M	48	2.73	1.512	0.439	110	0.662
		F	64	2.61	1.364			
Q31:When taking online classes, I would break down in the face of a large number of English tests	2.36	M	48	2.48	1.530	0.798	87.227	0.427
		F	64	2.27	1.212			
Q44:For me, learning English in the online mode is better for me to get high scores	2.68	M	48	2.73	1.198	0.384	110	0.702
		F	64	2.64	1.213			
Test anxiety	2.54	M	48	2.629	0.916	1.035	108	0.303
		F	64	2.466	0.754			

The interviews with the interviewees also illustrate this point. Interviewee F said, “exams are important, and I always feel nervous when taking exams.” Similarly, Interviewee H mentioned, “I am anxious about quizzes and exams because of the high expectations of my parents and the English teacher.” And Interviewee L said she was “overwhelmed by the heavy homework and the key points of review for exams, and even felt stressed out during the review.”

### Online class anxiety

As is shown in Table 8, the overall mean value of the students’ online class anxiety is 2.59. This indicates that Chinese rural junior high school students have a moderate level of test anxiety in English learning under the online class mode.

**Table 8:** Descriptive statistics of each question about online class anxiety.

Title	Mean	Gender	N.	Mean	SD	T	DF	Sig.
Q12:I feel that I have a good foundation in English and am able to navigate the online English learning classroom with ease	2.88	M	48	2.73	1.333	−1.113	83.903	0.269
		F	64	2.98	1.000			
Q13:I can memorize words independently, review what I have learned and preview what I will learn in a timely manner under the online class mode	3.01	M	48	2.92	1.334	−0.688	110	0.493
		F	64	3.08	1.145			
Q14:I can quickly adapt to the pace of English learning classes under the online mode	3.13	M	48	3.10	1.433	−0.202	110	0.840
		F	64	3.16	1.288			
Q21:During online English classes, my thoughts or attention is always drawn to other things	2.59	M	48	2.75	1.451	1.130	110	0.261
		F	64	2.47	1.181			
Q23:I can express my ideas well in online English classes	2.80	M	48	2.88	1.453	0.496	110	0.621
		F	64	2.75	1.208			
Q28:During online English classes, I feel nervous if I do not know the words or sentences I want to use	2.73	M	48	2.88	1.378	0.966	110	0.336
		F	64	2.63	1.339			
Q29:During the English online class, I would be flustered by the listening questions that I did not understand, otherwise I would be flustered	2.38	M	48	2.46	1.429	0.588	110	0.558
		F	64	2.31	1.194			
Q30:I feel pressured by every raw question in online English class	2.24	M	48	2.25	1.263	0.068	110	0.946
		F	64	2.23	1.165			
Q35:During online English classes, I feel nervous when my English teacher speaks English and I cannot understand every word	2.70	M	48	2.79	1.458	0.647	89.026	0.519
		F	64	2.63	1.189			
Q36:During online English classes, to avoid the teacher’s questions, I always used the excuse of poor network signal to excuse myself	1.77	M	48	1.85	1.130	0.759	110	0.449
		F	64	1.70	0.971			
Q38:The usual amount of homework in the online classroom mode is larger than that in the traditional classroom mode	2.39	M	48	2.58	1.485	1.285	86.806	0.202
		F	64	2.25	1.168			
Online class anxiety	2.59	M	48	2.655	0.597	1.082	109	0.282
		F	64	2.530	0.607			

Students do feel anxious about taking online English classes, according to the interviews. The following are some of the quotations from the interviewees. Interviewee I mentioned in her interview, “I feel inexplicably nervous and uneasy when taking online English classes, largely because of my poor pronunciation or inferiority.” And Interviewee K said, “lack of grammar knowledge and inability to form complete sentences make me anxious about taking English classes online.” And Interviewee J said, “I cannot keep up with the teacher during the online English classes; as a result, the classes always end in a daze.”

In the previous research, the subjects of foreign language anxiety research mainly focus on undergraduate students (Fong et al., 2022), graduate students (Tang, 2019) and international students (Cui, 2021), so that few studies involve primary and junior high school students. In this research, the first result investigated the moderate level of English learning anxiety of Chinese rural junior high school students under online class mode, broadening the scope of the research subjects.

### The relationship between gender difference and anxiety in online foreign language classes

The values of the 35 questionnaire questions for both male students and female students are shown in Table 9, and the scores fluctuate between 35 and 175. In Table 10, *p* = 0.317, (*p* > 0.05) revealed there is statistically no significant relation between gender difference and anxiety in online foreign language classes. However, there is statistically significant difference between male students and female students only in negative evaluation anxiety, and there is no statistically significant difference between male students and female students in communicative anxiety, test anxiety and online class anxiety, as is shown in Table 9.

**Table 9:** Descriptive statistics of each variable of English learning anxiety.

Anxiety variables	Gender	N.	Mean	SD	T	DF	Sig.
Communicative anxiety	M	48	2.763	0.751	1.285	110	0.202
	F	64	2.581	0.730			
Negative evaluation anxiety	M	48	2.595	0.743	2.064	110	0.041
	F	64	2.276	0.855			
Test anxiety	M	48	2.629	0.916	1.035	108	0.303
	F	64	2.466	0.754			
Online class anxiety	M	48	2.655	0.597	1.082	109	0.282
	F	64	2.530	0.607			
All anxiety	M	48	93.313	21.385	1.137	109	0.317
	F	64	86.813	22.741			

**Table 10:** Five strategies to relieve foreign language learning anxiety.

Strategy number	Strategies
1.	Recognizing the existence of anxiety correctly
2.	Communicating the anxiety honestly with others
3.	Improving the psychological quality
4.	Being positive about life’s setbacks
5.	Setting up some realistic goals in English learning

Table 5 shows that male students have a higher level of communicative anxiety than female students, for male students’ means of Q11, Q15, Q19, Q33, Q34, Q37, Q42, and Q43 are higher than those of female students, with the exception that male students’ means of Q18 and Q45 are slightly lower than those of female students. However, as is shown in Table 9, there is no statistically significant difference on each variable of communicative anxiety between male students and female students.

Table 6 shows that male students have a higher level of negative evaluation anxiety than female students, for male students’ means of Q16, Q20, Q22, Q26, Q27, Q32, Q39, Q40, and Q41 are higher than those of female students. However, as is shown in Table 9, there is statistically significant difference between male students and female students only in Q22 and Q 39.

Table 7 shows that male students have a higher level of test anxiety than female students, for male students’ means of Q17, Q24, Q25, Q31, and Q44 are higher than those of female students. However, as is shown in Table 9, there is no statistically significant difference on each variable of test anxiety between male students and female students.

In Table 8, male students have a higher level of online class anxiety than female students, for male students’ means of Q21, Q23, Q28, Q29, Q30, Q35, Q36, and Q38 are higher than those of female students, with the exception that male students’ means of Q12, Q13, and Q14 are slightly lower than those of female students. However, as is shown in Table 9, there is no statistically significant difference on each variable of online class anxiety between male students and female students.

Traditionally in China, female students are usually perceived as superior to male students in terms of language learning competence, particularly foreign language learning competence. Females usually perform better than males in English exams. Therefore, it is easy to imagine that females have more confidence than males in learning English well. Once they gain confidence in their ability, they will be more comfortable in dealing with difficulties in English learning. On the contrary, males are in an extremely active state physically and mentally at this time in junior high school and are so interested in new things around them that they tend to neglect learning in the language. Males suffer more failures in English, so they tend to attribute their failures to their low language learning skills. As a result, they become anxious about English classes, especially online English classes.

What’s more, Table 9 shows that male only has higher communication anxiety than female, however, there is statistically no significant relation between gender difference and all anxiety in online foreign language classes in Table 9, which is consistent with the research of Garcia de Blakeley et al. (2017).

### Sources of foreign language learning anxiety for Chinese rural junior high school students

Through analysis of the questionnaires and interviews, it was found that the sources of English learning anxiety of Chinese rural junior high school students include the students themselves, their home environments, the teacher and the school, and the social environment, shown in Table 11.

**Table 11:** Sources of English learning anxiety.

	Home environment	Students themselves	Teacher and School	Social environment
Mean	1.619	2.636	2.381	2.607
SD	0.322	0.647	0.824	0.840

Among the four major items exploring the sources of anxiety, the mean value of the item of students themselves was the highest, which indicates students themselves are one of the main sources of anxiety. On the one hand, students’ lack of English knowledge and inferiority are the main sources of their anxiety. In Table 6, Q20 has a mean score of 3.00 for males and 2.89 for females, which indicates that many students feel nervous when they can’ t understand what the teacher says or means in online classes, which is a sign of their lack of confidence and is not conducive to learning new knowledge efficiently in online classes. On the other hand, the habit of students seldom using English knowledge will make students anxious when they encounter problems. The average score of Q31 in Table 7, is in the middle level. From the interviews with students, we know that the knowledge they learn in class is not well applied to the usual practice and tests. Even though they put in more effort than students with good test scores, they still do not know how to do the test with a large number of questions, which causes confusion and anxiety about learning English in the long run.

Of the four major items that were used to explore the sources of anxiety, the mean value of home environment was the lowest, as is shown in Table 11. The overwhelming majority of interviewees said in the interviews, “my family fully support the online class mode, so nothing disturbs me during the online English classes,” but “occasionally the Internet speed is a little slow during the online class, and I cannot hear very clearly what the teacher says,” as Interviewee F mentioned. Here we can probably conclude that most of the home environment in the online class mode helps students learn English positively and does not make them feel nervous, confused, or even anxious.

After analyzing the questionnaires, it was found that the mean rank of teacher and school item reached 2.381, ranking the third. For Q24 in Table 7, the mean of 3.02 for males and 2.84 for females show that the majority of students are worried and concerned about their test scores, both in the offline classroom and in the online mode of English learning. The subjects were first-year students. Compared with the high school level of learning, the number of subjects in junior high school has changed, and the attention to the subject of English will decrease, but middle school English requires students to start building a solid foundation, and also increases the ability to listen, read, and write, which increases the overall difficulty. Students with a mediocre foundation will feel overwhelmed by learning all of a sudden, and thus cannot adapt to the rhythm of online classes, as one of the main subjects English test scores, one of the main subjects, put more pressure on students and create a sense of tension and anxiety. This is well reflected in Q41, where the mean value is 2.60 for males and 2.34 for females, and it is true that teachers cannot take care of each student’s specific situation when teaching online. Some students may worry that their situation will not be solved well, which may lead to anxiety.

The mean value of social environment is second only to their own problems, with a mean value of 2.607 (Table 11). The mean value of Q42 reached 2.54 (Table 5), indicating that the general environment of the pandemic has a certain influence on students’ English learning. Teachers and students have to change their teaching and learning methods, which is a great challenge for both teachers and students.

The data from the questionnaires concluded that the causes of tension and even anxiety are family environment, their own sources, teachers’ and school’s factors, and social environment. The results of the interviews revealed that most of the students also think so, and their own factors and social environment are the main factors that affect junior high school students’ anxiety in learning English online. This coincides with the information from the questionnaire data. Moreover, low self-evaluation of students was found to affect online classroom anxiety of rural junior high school students in this result, which echoes the finding of Zhang and Guo (2018) who found low self-efficacy led to high foreign language anxiety. Besides, Gkonou (2017) and Saghafi et al. (2017) proved the influence of contextual factors on foreign language anxiety of students, but no details have been given. This research further complemented that home and school environment were important contextual factors influencing foreign language anxiety in the third research objective.

### Strategies to relieve foreign language learning anxiety for Chinese rural junior high school students

In order to reduce the negative effects of English learning anxiety on junior middle school students in online classes, the interviews were transcribed and encoded to explore the following strategies to relieve foreign language learning anxiety for Chinese rural junior high school students in Table 10.

As Table 10 shown, junior high school students firstly should have a correct understanding of their own tension or anxiety, and should realize that it is a normal phenomenon and everyone has more or less tension and anxiety. Students should face up to their anxiety instead of avoiding it or treating it negatively.

Secondly, talking to someone about their anxiety and asking for help are effective ways to relieve it. If students are willing to share their emotions, they can confide in their teachers and classmates more often. While others help, the most important thing is to improve their own psychological quality.

Thirdly, good psychological quality is an indispensable factor of English learning for students with poor foundation. Students should overcome their inferiority complex, develop their self-confidence, and participate in classroom activities actively and bravely.

What’s more, it is important to face up to the setbacks in life. In the general environment of the pandemic, we cannot change the environment of learning, so they should receive the existing environment positively and optimistically and keep an optimistic attitude towards English learning.

Finally, it’s strongly advised setting up some realistic goals in English learning. In order to make progress in English learning in the online mode or in the usual study, it is very necessary to establish realistic goals. Initially, goals should be set from their own reality, and they should be moderate, not too high or too low, so that students can have enough motivation and confidence to achieve their goals. There should be both long-term and short-term goals. When short-term goals are achieved, students will have confidence in themselves and will be more motivated to achieve their long-term goals. In this way, the tension and anxiety will be significantly reduced when the English learning process is based on goals step by step.

In the past research, Miyazoe and Anderson (2011) put forward that anonymous speech can effectively relieve anxiety in online class, and Fong et al. (2022) pointed out that repeated practice can reduce speech anxiety in foreign language class, however, none of them discussed how to reduce students’ own psychological anxiety from the perspective of cognitive psychology. Therefore, the last result summarized five strategies to relieve foreign language learning anxiety, including recognizing the existence of anxiety correctly, communicating the anxiety honestly with others, improving the psychological quality, being positive about life’s setbacks, and setting up some realistic goals in English learning, which provides the reference for scholars to further explore ways to reduce foreign language anxiety from cognitive and psychological perspectives.

## Conclusion

The research found that Chinese rural junior high school students have a moderate level of English learning anxiety under the online class mode, among which students’ communicative anxiety is the highest, followed by online class anxiety, test anxiety and negative evaluation anxiety. Besides, Chinese rural junior high school male students have slightly higher level of English learning anxiety than female students under the online class mode. However, there is statistically significant difference between male students and female students only in negative evaluation anxiety, and there is no statistically significant difference between male students and female students in communicative anxiety, test anxiety and online class anxiety. Additionally, four factors that affect online foreign language anxiety were found, including the students themselves, their home environments, the teacher and the school, and the social environment. Lastly, five strategies to relieve foreign language learning anxiety are also discussed, including recognizing the existence of anxiety correctly, communicating the anxiety honestly with others, improving the psychological quality, being positive about life’ s setbacks, and setting up some realistic goals in English learning.

To sum up, the research has four major implications. First of all, this research takes rural junior high school students as participants, expanding the scope of research subjects on online foreign language classroom anxiety. Secondly, from a quantitative perspective, this study proves that gender has no significant effect on anxiety in online foreign language classes, providing material for research on the relationship between gender and anxiety. In addition, the research on the factors of online foreign language anxiety provides a supplement to previous relevant studies. Finally, the strategies for relieving anxiety proposed in this research from the cognitive and psychological perspectives, providing a research perspective for future scholars.

However, there are still some limitations in the present study, for example, this study only discussed the influence of online foreign language classroom anxiety from the perspective of students. Due to time and resource limitations, 120 Chinese rural junior high school students were selected to conduct the questionnaires, and 12 to conduct the interviews. If future studies include teachers’ samples, the research may be more convincing and reliable.

## Data availability statement

The original contributions presented in the study are included in the article/supplementary material, further inquiries can be directed to the corresponding author.

## Ethics statement

Ethical review and approval was not required for the study on human participants in accordance with the local legislation and institutional requirements. The participants provided their written informed consent to participate in this study.

## Author contributions

QY designed the study, interpreted the data, and wrote and revised the manuscript. DX analyzed the data and revised the manuscript. RH conducted the questionnaire survey and interview survey, analyzed the data, and helped with the first draft of the manuscript. All authors contributed to the article and approved the submitted version.

## Funding

This work was supported by grants from the Research Project of Humanities, Foreign Languages and Arts, Xi’an University of Technology (2020RY011).

## Conflict of interest

The authors declare that the research was conducted in the absence of any commercial or financial relationships that could be construed as a potential conflict of interest.

## Publisher’s note

All claims expressed in this article are solely those of the authors and do not necessarily represent those of their affiliated organizations, or those of the publisher, the editors and the reviewers. Any product that may be evaluated in this article, or claim that may be made by its manufacturer, is not guaranteed or endorsed by the publisher.
